# Comparing different technologies for active TB case-finding among the homeless: a transmission-dynamic modelling study

**DOI:** 10.1038/s41598-018-19757-5

**Published:** 2018-01-23

**Authors:** Tendai Mugwagwa, Helen R. Stagg, Ibrahim Abubakar, Peter J. White

**Affiliations:** 1 Modelling and Economics Unit, National Infection Service, Public Health England, London, UK; 2 MRC Centre for Outbreak Analysis and Modelling, and NIHR Health Protection Research Unit in Modelling Methodology, Department of Infectious Disease Epidemiology, Imperial College London, London, UK; 3 Institute for Global Health, Faculty of Population Health Sciences, University College London, London, UK; 4 Medical Directorate, Public Health England, London, UK

## Abstract

Homeless persons have elevated risk of tuberculosis (TB) and are under-served by conventional health services. Approaches to active case-finding (ACF) and treatment tailored to their needs are required. A transmission-dynamic model was developed to assess the effectiveness and efficiency of screening with mobile Chest X-ray, GeneXpert, or both. Effectiveness of ACF depends upon the prevalence of infection in the population (which determines screening ‘yield’), patient willingness to wait for GeneXpert results, and treatment adherence. ACF is efficient when TB prevalence exceeds 78/100,000 and 46% of drug sensitive TB cases and 33% of multi-drug resistant TB cases complete treatment. This threshold increases to 92/100,000 if additional post-ACF enhanced case management (ECM) increases treatment completion to 85%. Generally, the most efficient option is one-step screening of all patients with GeneXpert, but if too many patients (>27% without ECM, >19% with ECM) are unwilling to wait the 90 minutes required then two-step screening using chest X-ray (which is rapid) followed by GeneXpert for confirmation of TB is the most efficient option. Targeted ACF and support services benefit health through early successful treatment and averting TB transmission and disease. The optimal strategy is setting-specific, requiring careful consideration of patients’ needs regarding testing and treatment.

## Introduction

In England in 2015, 5,758 TB cases were notified^1^, of which 4.4% had a history of homelessness^1^. In that year, the prevalence of resistance to at least rifampicin (RIF) or both RIF and isoniazid (hereafter referred to as MDR TB) was 3.6% in the TB disease of the homeless, compared to England’s national average of 1.6%^1^. Recently, the Collaborative Tuberculosis Strategy for England highlighted the need to improve access to health services, ensure early TB diagnosis, and tackle MDR TB in under-served groups^2^. In low TB burden countries, appropriately targeted interventions such as active case finding (ACF) can be effective in controlling TB^3,4^. The use of a mobile X-ray unit (MXU) successfully reduced TB transmission in Paris^5^ and Rotterdam^6^.

Approaches to TB control need to be effective and efficient. There are several possible approaches to screening using different technologies in a mobile unit: chest X-ray (CXR) followed by referral to hospital for diagnosis and treatment if appropriate; GeneXpert (GX) MTB/RIF, which diagnoses TB in the mobile unit followed by referral to hospital for TB treatment; or CXR followed by GX on the mobile unit followed by referral to hospital for TB treatment for those with abnormal CXR and positive GX results. GeneXpert can detect *Mycobacterium tuberculosis* (MTB) and RIF resistance, a marker for MDR TB (since RIF resistance is typically associated with resistance to isoniazid), with high sensitivity in about 90 minutes^7^, and has been shown to be beneficial in TB control^8–10^. Adherence to TB treatment is often difficult for homeless persons; treatment completion in the absence of assistance has been recorded as 46% for drug sensitive (DS) TB cases among the homeless in London^11^.

Using an integrated transmission-dynamic and health-economic mathematical model we evaluated different screening approaches, considering the resource requirements and benefits relating not only to the individuals who are screened and (where appropriate) treated but also to the population through averting TB transmission. We considered different strategies for case-finding and diagnosis and examined how the prevalence of infection and MDR TB affects effectiveness and efficiency.

## Methods

### Model description

**Figure 1:**
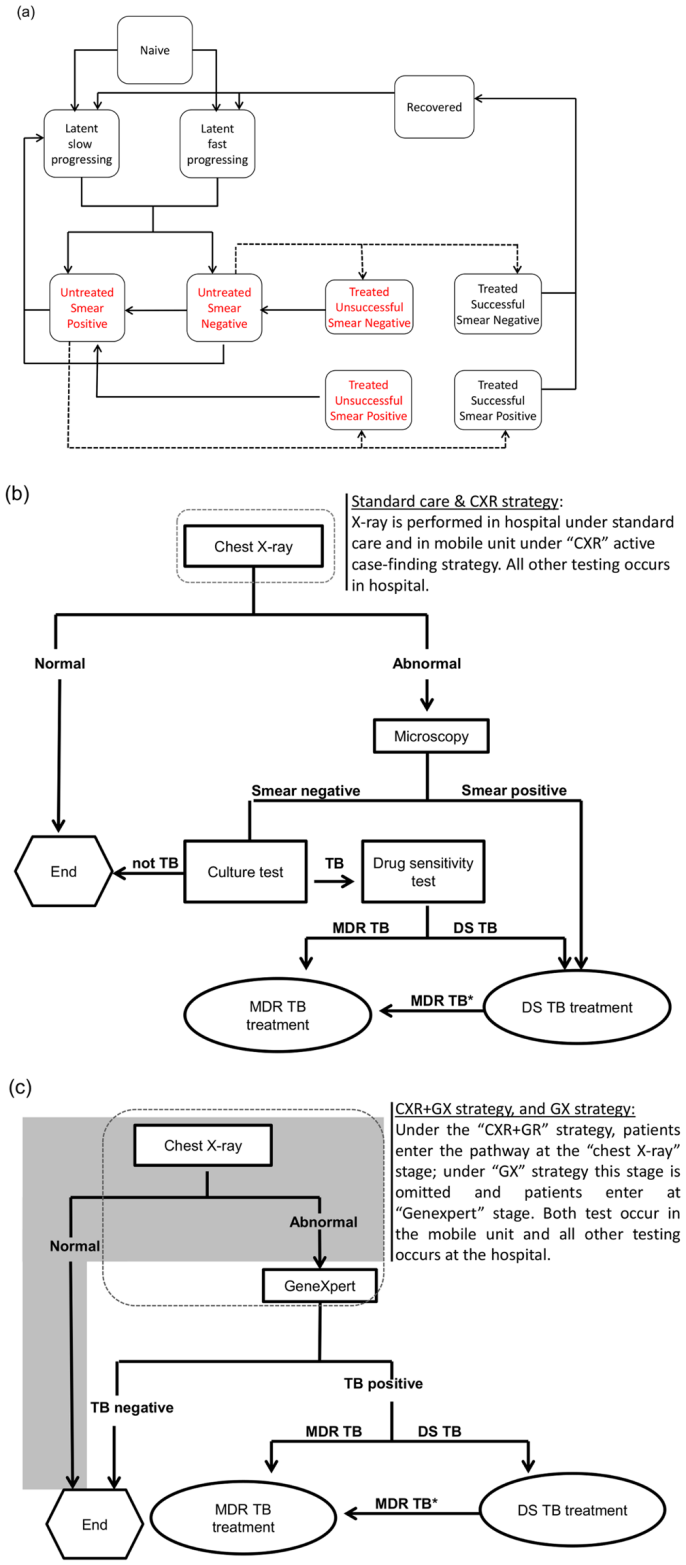
Flow diagram showing health states, treatment outcomes and the passive and active case-finding care pathways followed by patients in the compartmental model of tuberculosis. (**a**) Compartments labelled in red denote infectious health states. Dashed lines represent the process of diagnosis and treatment initiation, following pathways represented in (**b**) and (**c**). Panel (**b**) shows the radiology based case finding pathway and (**c**) the radiology followed by GeneXpert case finding pathway. MDR TB* disease presumptively treated with DS TB drugs switched to MDR TB treatment after DST results.

We developed a mathematical model describing pulmonary TB transmission and disease progression in a population of homeless persons (Fig. 1a), parameterised from the literature^1,7,11–31^. Individuals enter the population through becoming homeless and exit from the population through ceasing to be homeless or death (natural or TB associated). Individuals entering the homeless population are assumed to be uninfected, as prevalence in the general population is extremely low. Individuals who cease to be homeless re-enter the general population, with those who acquired TB infection whilst homeless still being at risk of progressing to active disease and requiring treatment. This enables us to capture the benefits of reducing TB transmission in the homeless population in averting illness in people who cease to be homeless. We vary the prevalence of active TB from 10 to 2000 per 100,000, and the proportion of infections that are MDR TB from 3% to 15%.

The model’s representation of the natural history of TB and effects of treatment are shown in Fig. 1. Uninfected individuals who acquire either DS TB or MDR TB infection develop either fast-progressing or slow-progressing latent infection; the latter can be subject to exogenous re-infection, leading to fast-progressing latent infection. They progress to active disease which is either sputum smear-negative or smear-positive, with the latter being more infectious^32^. Smear-negative disease can progress to become smear-positive. In the absence of treatment individuals can revert to latent infection due to natural immune processes.

Following diagnosis (Fig. 1), patients begin treatment, with a proportion being adherent and completing treatment and the remainder not doing so. The duration of completed treatment is longer for MDR TB. Successfully treated patients are rendered non-infectious whilst on treatment^33^, and upon completion enter a recovered state in which they have partial immune protection against subsequent infection. Non-adherent patients remain infectious whilst on treatment and upon loss to follow up return to their previous disease state. Inappropriately treated adherent patients can have their treatment corrected after obtaining drug sensitivity test results (DST).

For simplicity, we consider two TB strains in circulation, drug sensitive (DS) TB and MDR TB. The resource requirements and duration of MDR TB treatment is significantly higher than drug sensitive and mono-resistant TB. In the model MDR TB arises in DS TB infected patients who are non-adherent to treatment (acquired resistance) at a rate of 0.035 per year. MDR TB is also transmitted by patients who are already infected by MDR TB (primary resistance)^34^. The MDR TB transmission rate is fitted for each scenario to produce the required % MDR TB at baseline.

The average duration of symptoms prior to presenting to healthcare is 121 days and the diagnostic delay until starting treatment is 16 days for smear negatives and 1 day for smear positives^11^. Patients who are sputum smear-positive are presumptively started on DS TB treatment prior to receiving culture analysis and DST results. MDR TB cases switch to appropriate treatment once the TB culture and DST results are obtained. Smear-negative cases face a delay of up to 8 weeks^35^, for culture-based TB confirmation and DST results before starting treatment, during which time they may be lost to follow up before treatment initiation. Treatment of drug-sensitive TB takes 6 months and MDR TB treatment takes 20 months^13^. Patients on inappropriate treatment remain fully infectious regardless of adherence and are not cured^36^. In scenarios where GeneXpert is used, false positive results could lead to unnecessary treatment for TB or treatment for MDR TB when the infection is drug sensitive, which will be rectified as part of clinical management following culture and DST results.

Patients who self-present to health services (“passive case-finding”) are screened using chest X-ray (Fig. 1b), and patients with abnormal radiology are offered sputum smear microscopy and culture testing. Smear-positive patients are treated for TB (which is presumed to be DS, with the regimen changed if drug resistance is detected by culture); smear-negative patients are treated if they receive a positive culture result.

In the model we compare intervention strategies, starting each time with a steady-state population (with passive case-finding) prior to the intervention (parameter values summarised in Supplementary Table S1). The mobile screening unit implements each of the following ACF strategies (summarised in Table 1 and Fig. 1b,c): CXR: screening by chest X-ray, with cases of abnormal radiology referred to hospital for diagnosis; GX: screening by GeneXpert, with TB (and MDR TB) diagnosed on the mobile unit, and if TB is diagnosed then patients are referred to hospital for treatment; CXR + GX: initial screening by chest X-ray, with cases of abnormal radiology tested by GeneXpert on the mobile unit, and diagnosed cases of TB referred to hospital for treatment. In all cases patients referred to hospital are given assistance, which requires staff time. Rates of mobile screening were based on Jit *et al*.^11^ and adjusted for the performance of different testing technologies. For the GX option, in which all patients are screened by GeneXpert, a 16-channel GeneXpert machine is required, whilst for the CXR + GX option a 4-channel machine is sufficient. As homeless patients often require assistance in adherence to treatment, we calculated a threshold resource requirements for enhanced case management (ECM) which increases the proportion of diagnosed patients successfully completing treatment to the WHO target level of 85%^37^, for both DS TB and MDR TB: if ECM can be provided for less resource per patient than this threshold then it would be considered favourable.

**Table 1:** Summary of screening, diagnosis and treatment management strategies considered. Note that the table indicates the tests (except for the smear test which occurs at the hospital) performed on the mobile unit before referral of appropriate patients to hospital for diagnosis/treatment.

Screening strategy	Tests performed by mobile unit		TB diagnosis location	Tests performed in clinic		Enhanced case management for treatment
	X-ray	GeneXpert		Sputum smear	Culture	
CXR	Yes		Clinic	Yes	Yes	
CXR + ECM	Yes		Clinic	Yes	Yes	Yes
GX		Yes	Mobile unit		Yes	
GX + ECM		Yes	Mobile unit		Yes	Yes
CXR + GX	Yes	Yes	Mobile unit		Yes	
CXR + GX + ECM	Yes	Yes	Mobile unit		Yes	Yes

Unit costs and health utility values (measured in QALYs) are given Supplementary Table S2 and Supplementary Table 3. Prices were adjusted to 2015–16 values using the hospital & community health services (HCHS) index^25^. Future resource requirements and health benefits were discounted at a rate of 3.5% per annum as recommended for the UK by the National Institute for Health and Care Excellence (NICE)^14^. Cumulative discounted resource requirements and health outcomes were calculated for the lifetime of the initial cohort (100 model simulation years). The incremental net benefit (INB) of an intervention is calculated as follows. The incremental health benefit (compared with baseline) is multiplied by the monetary value of a unit of health. In the UK, health is measured in QALYs. In the UK a QALY is typically valued at £20,000 – £30,000 and it is usual practice to use both values. The incremental resource requirement of an intervention is subtracted from the monetary value of the incremental health benefit to derive the INB. If the INB is positive then the intervention is more favourable than the comparator.

There is a lack of information on the resource requirements of providing effective ECM, so we calculated the maximum daily requirement for providing ECM to a patient on treatment below which the whole intervention package would be considered efficient, considering the benefits of ECM in terms of its effects on other net resource requirements and health (QALYs), with a QALY valued at £20,000 or £30,000. We calculated the net health benefit and net resource saving accruing from increasing the treatment completion rate from 46% for DS TB cases and 33% of MDR TB cases to the target of 85%, and then calculated the corresponding monetary value if a QALY were valued at £20,000 or £30,000.

A potential difficulty in using GeneXpert in the mobile unit is loss to follow-up of patients awaiting their test result, which takes around 90 minutes. A study of patients seeking testing for sexually-transmitted infections found that most would not even wait 30 minutes for the results of a test that they had sought and travelled to obtain^38^, whereas the TB screening service approaches individuals and invites them to be tested for an infection that they probably do not have. This will be particularly challenging if GeneXpert were used to screen all patients, meaning that a large number of patients would have to have long waits. Patients lost to follow up whilst awaiting the GeneXpert result cause testing resources to be used for no health benefit (to the patient or the population, from averted transmission) or resource savings (from averted transmission). We examine how varying the proportion of patients lost to follow-up during GX screening affects the relative efficiency of the screening options without ECM and with ECM.

### Parameter estimation and model fitting

Demographic parameter values for the modelled homeless population were based on the Combined Homelessness and Information Network (CHAIN) database^12^. We considered a range of DS and MDR TB prevalence that encompasses values reported elsewhere^15,39^. TB transmission model parameter values were either obtained from literature^11,16,17^, or estimated by model fitting. TB case-finding rates by mobile radiology screening were based on Jit *et al*.^11^. The model was implemented in Python version 2.7 using the Forward Euler method and fitted to prevalence on treatment and proportion MDR TB using Python’s ‘leastsq’ algorithm. Homeless settings with varying TB burden and demography were considered. Details of model parameter values are shown in Supplementary Table S1.

### Sensitivity analysis

We performed one-way sensitivity analyses varying screening and diagnostic accuracy parameters ± 20% around their posterior means for the baseline population. The rest of the model parameters were fixed (values given in Supplementary Table S1, Supplementary Table S2 and Supplementary Table S3). Incremental net benefit was evaluated for each strategy in Table 1 and a ranking assigned relative to the rest of the strategy grouping with or without ECM.

## Results

### Impact of ACF over time

**Figure 2:**
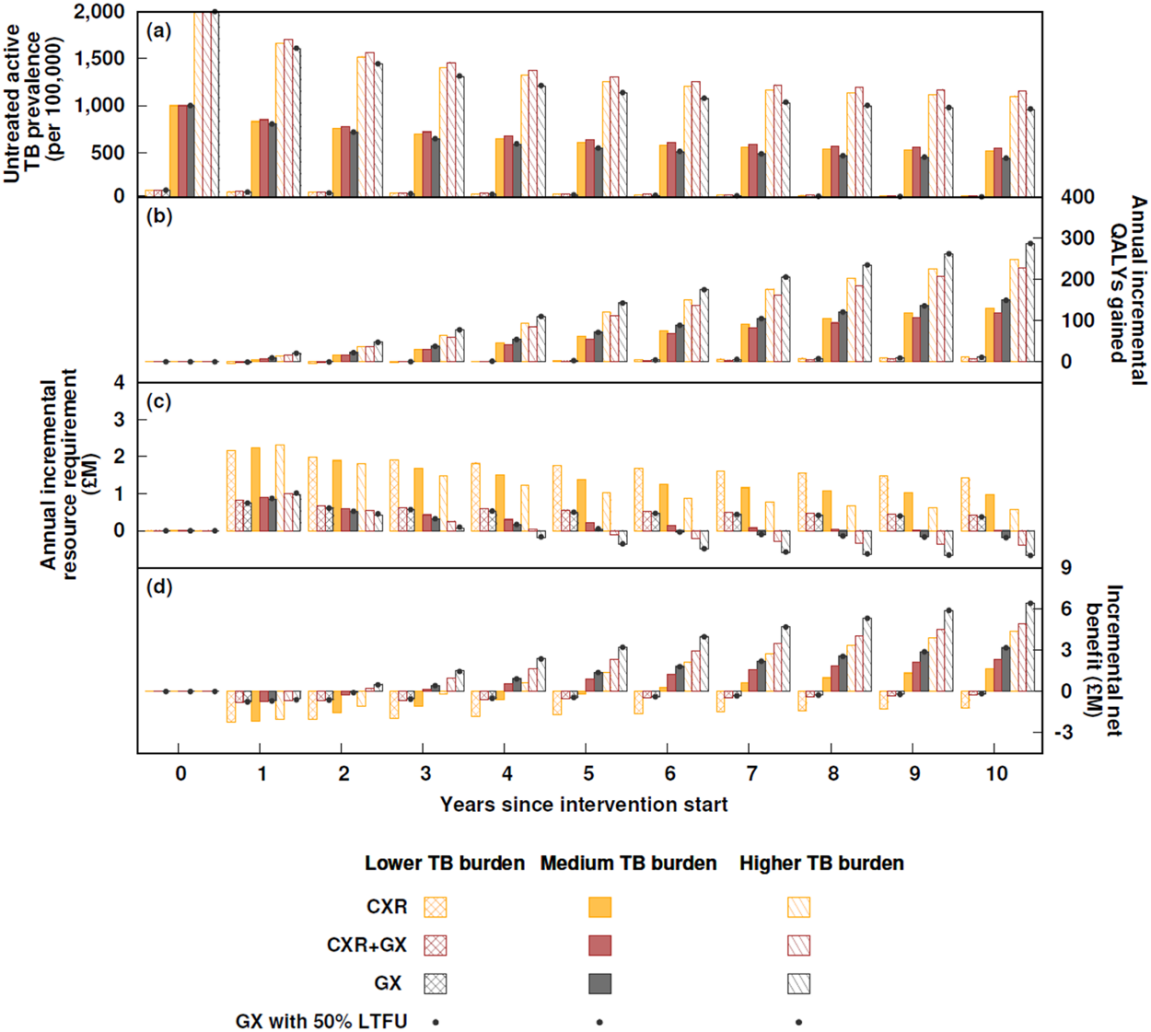
Impact of different active case-finding strategies on TB prevalence, resource requirements, QALYs and overall efficiency over time in settings with different TB burden. (**a**) Untreated active TB prevalence, (**b**) annual incremental resource requirement, (**c**) annual incremental QALYs, (**d**) annual incremental net benefit in settings with a TB burden that was lower (initial prevalence 100 per 100,000), medium (1,000 per 100,000) and higher (2,000 per 100,000) settings. The proportion of untreated active MDR TB was fixed at 7.5%, the mid-point of the range considered in this paper. The treatment completion rate is set at 46% for drug-sensitive TB cases and 33% of MDR TB cases. Time zero indicates the starting conditions, before ACF is introduced. For GeneXpert, the bars represent the scenario with no loss to follow-up whilst awaiting the test result; the effect of 50% loss to follow-up is shown by the dots.

All ACF strategies reduced the burden of active TB (Fig. 2a) by identifying and treating cases earlier than passive case-finding alone and therefore reducing the prevalence of untreated active TB, which also averts onward transmission, thus reducing incidence (Supplementary Figure S1 and Supplementary Figure S2). Figure 2a shows how the ACF with CXR, GX, and CXR + GX affects TB dynamics in settings with three different TB burdens that are “higher” (initial prevalence 2,000 per 100,000), “medium” (1,000 per 100,000) and “lower” (100 per 100,000) for homeless populations. As would be expected, the greatest impact in reducing transmission and disease was in the higher TB burden setting and the least impact in the lower-burden setting (Fig. 2a).

The GX and CXR + GX options result in fewer referrals to hospital than CXR option. This reduces resource requirements of assistance in referral. However, the GX option requires that all patients have a long waiting time (GeneXpert takes about 90 minutes), whereas with the CXR and CXR + GX options patients receive their X-ray result quickly. Those with normal radiography therefore have no further wait, whilst those with abnormal radiography are assumed to have a motivation to wait for the GeneXpert result to discover if the abnormality is due to TB.

Over time the prevalence of active TB declines due to a reduction in the average duration of illness prior to diagnosis and treatment, and due to a reduction in the incidence of infection resulting from the reduction in prevalence. Health, measured in Quality-Adjusted Life-Years (QALYs), is gained in the population through averting death due to TB, and averting morbidity due to TB and improved health associated with TB treatment (Fig. 2b). The resource needs of the programme are highest in the first year (Fig. 2c), partly due to setting-up and partly because the highest rates of treatment occur in the first year due to the prevalence of active TB being highest; subsequently the need for treatment declines due to incidence decreasing.

The changing patterns over time in the health benefits and resource requirements of the programme mean that the incremental net benefit (INB; calculated for the lifetime of the initial cohort, 100 model simulation years, by expressing incremental health benefits in monetary terms and subtracting resource requirements, as explained in the Methods section) is lowest in the first year and then increases over time (Fig. 2d). Where the INB is negative the programme would be considered unfavourable and where it is positive the programme would be considered favourable. This means that the time period considered in the analysis is important: the annual INB only becomes positive in the higher-burden setting in year 2 or 3 (depending on the strategy) and in the medium-burden setting in years 2–5; the longer the time-period that is considered the more favourable the programme because the health benefits are greater in later years whilst the resource requirements are lower. Importantly, in the lower burden setting, the annual INB only becomes positive in year 11 (not shown).

### Efficiency of ACF strategies in settings with different prevalences of TB and MDR TB

**Figure 3:**
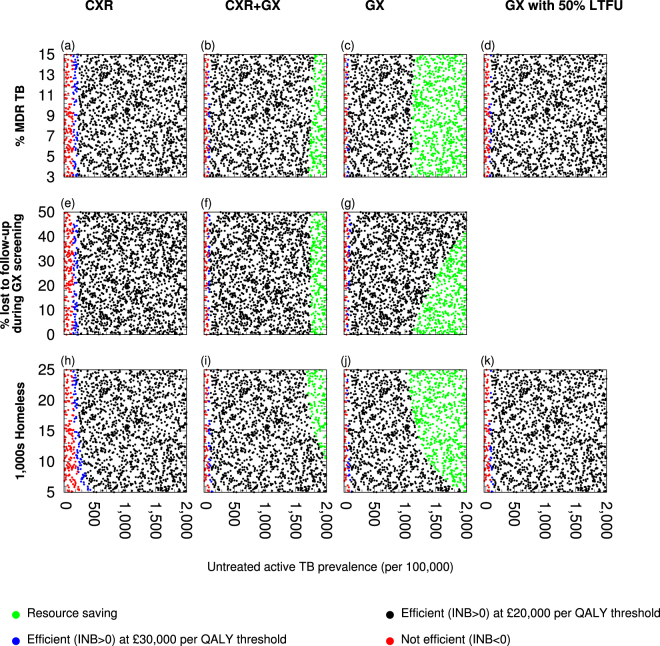
Efficiency of different active case finding (ACF) strategies without enhanced case management (ECM) in different settings. Different ACF strategies were compared with current practice in different settings in which the prevalence of untreated active TB prior to intervention was varied, along with (**a**–**d**) the percentage of multi-drug/rifampicin resistant (MDR) TB among the untreated active TB cases, (**e**–**g**) the proportion of individuals lost to follow-up whilst awaiting GeneXpert results or (**h**–**k**) the homeless population size. The incremental net benefit of each strategy without ECM was calculated over the lifetime of the initial cohort. Panels (**a**–**c**) and (**h**–**j**) represent scenarios in which all individuals screened with GeneXpert wait for their results (no loss to follow-up (LTFU)); panels (**d**) and (**k**) represent scenarios with 50% LTFU.

We examined how the prevalence of TB and specifically MDR TB affects the efficiency of interventions by varying those prevalences (Fig. 3a–d). For each intervention option there is a minimum prevalence below which screening is not favourable even if a QALY were valued at £30,000; there is a range of prevalence in which screening would be favourable if a QALY were valued at £30,000 but not £20,000; finally, there is a prevalence above which screening would be favourable if a QALY were valued at £20,000.

When TB prevalence is high, screening is more likely to be efficient (Fig. 3a–d) due to the greater yield of screening and averting more TB transmission and consequent active TB disease (Supplementary Figure S1). At a low TB prevalence (below 78 per 100,000), the number of TB cases averted was low and reductions in future resource requirements were lower than intervention resource requirements, and the positive predictive value (PPV) of screening was low, resulting in a high proportion of false positives which incur resource requirements of referral, and potentially treatment, without a health benefit. As TB burden increases, PPV increases, as do the resource savings and health benefits of averting more TB disease relative to intervention resource requirements.

**Figure 4:**
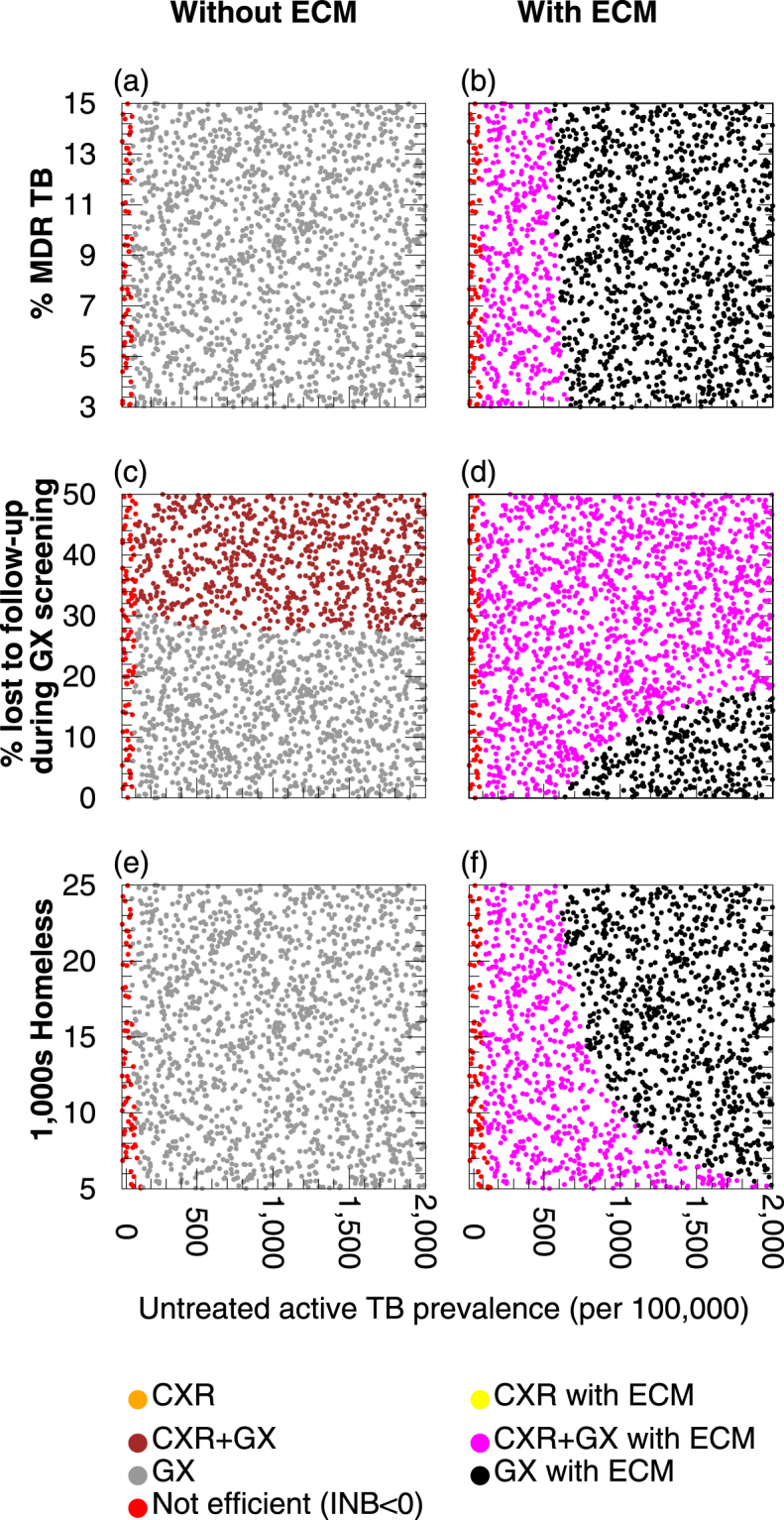
The most efficient active case finding (ACF) strategy in different settings. A summary of the most efficient ACF strategy based on (**a**,**c**,**e**) incremental net benefit of each strategies without treatment management and (**b**,**d**,**f**) maximum threshold resource requirements for enhanced case management for strategies in different population setting. Different ACF strategies were compared with current practice in different settings in which the prevalence of untreated active TB prior to intervention was varied, along with (**a**,**b**) the percentage of multi-drug/rifampicin resistant (MDR) TB among the untreated active TB cases, (**c**,**d**) the proportion of individuals lost to follow-up (LTFU) whilst awaiting GeneXpert results or (**e**,**f**) the homeless population size. Default parameter values in Supplementary Table S1, Supplementary Table S2 and Supplementary Table S3 were used. The “INB < 0” result indicates that no option had a positive INB with a QALY valued at £20,000. Panels (**a**,**b**) and (**e**,**f**) represent scenarios in which all individuals screened with GeneXpert wait for test results (no LTFU).

Across the ranges of TB burden and MDR TB proportions, where screening was favourable the most efficient strategy was the GX option – if patients are willing to wait the 90 minutes required for the test (Fig. 4a). This is examined further, below.

### How much is it worth spending on enhanced case management?

**Figure 5:**
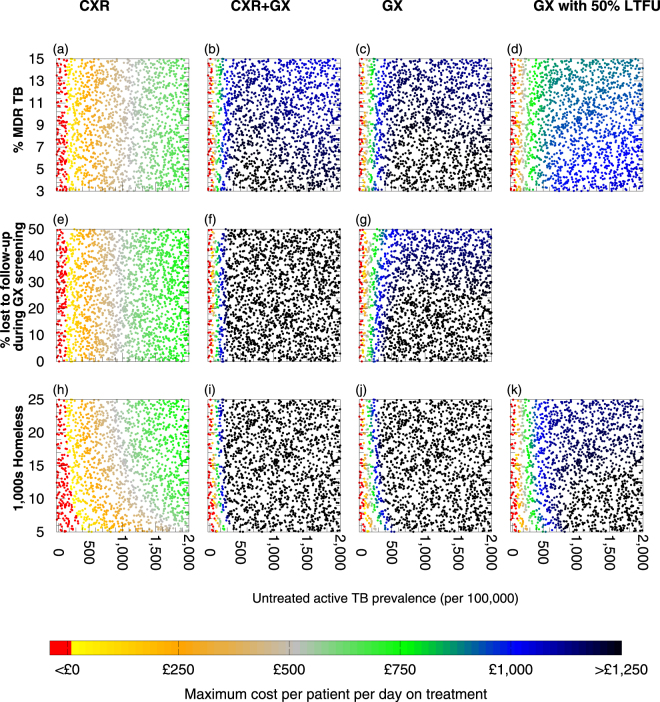
A summary of the maximum cost for enhanced case management (ECM) that would be considered favourable in different settings. Different ACF strategies were compared with current practice in different settings in which the prevalence of untreated active TB prior to intervention is varied, along with (**a**–**d**) the percentage of multi-drug/rifampicin resistant (MDR) TB among the untreated active TB cases, (**e**–**g**) the proportion of individuals lost to follow-up whilst awaiting GeneXpert results or (**h**–**k**) the homeless population size. The cost for enhanced case management per individual per day on treatment is calculated for each strategy with ECM with a QALY valued at £20,000. Above this threshold, the ACF strategy would not be unfavourable. Red indicates that ACF is not favourable even if treatment completion of 85% occurred without ECM being required. Panels (**a**–**c**) and (**h**–**j**) represent scenarios in which all individuals screened with GeneXpert wait for test results (no loss to follow-up (LTFU)); panels (**d**) and (**k**) represent scenarios with 50% LTFU.

Providing enhanced case management (ECM) to patients to increase treatment completion has benefits for the health of the average treated individual (increasing the health benefit obtained per person diagnosed) and reduces onward transmission (Supplementary Figure S1 and Supplementary Figure S2), both of which increase overall efficiency – provided that ECM is not too resource-intensive (Fig. 5a–d). There is a maximum cost of providing ECM per patient per day above which ECM would not be considered favourable. This maximum cost depends upon the cost savings (in £) occurring as a result of ECM, the health benefits (in QALYs) resulting from ECM, and the monetary value of a QALY (which allows health gains in QALYs to be converted to a value in £). If the resource requirements of ECM per patient per day were more than this threshold then it would not be considered favourable, whilst if ECM required less than this threshold then it would be considered favourable (Fig. 5a–d). The particular value of the threshold for ECM to be considered favourable depends upon the setting. In settings with higher rates of TB transmission and higher proportions of TB that is MDR there is a greater benefit of successful treatment because a greater amount of transmission is then averted, producing greater health benefits, as well as greater resource savings from averting future cases of illness that would require treatment. GX with ECM – provided patients waited for their test results – was the most efficient strategy for ACF (Fig. 4b).

### Loss to follow-up whilst awaiting GeneXpert results

Considering the potential problem of loss to follow-up of patients awaiting their GeneXpert test result, we examine how varying the proportion of patients lost to follow-up during GX screening affects the relative efficiency of the screening options without ECM (Fig. 3e–g) and with ECM (Fig. 5e–g). If patients are lost to follow-up whilst awaiting GeneXpert results for GX and CXR + GX options then the GX option is the most efficient. However, it is likely that patients with an abnormal chest X-ray will wait for the GeneXpert result to determine the cause of the abnormality, and since only a small number of patients require the second step they can be accommodated on the van whilst waiting. If there is no loss to follow-up with the CXR + GX option, then it is more efficient than the GX option if the GX option’s loss to follow-up rate is > 27%, in the absence of ECM for those treated for TB (Fig. 4c,d). If ECM for treatment is provided then if the untreated TB burden is > 640 per 100,000 and the proportion of patients lost to follow-up in GX screening is < 19% then the GX + ECM option may be more efficient than CXR + GX + ECM (Fig. 4d); but note that in this region of the graph there is a trade-off of untreated TB prevalence vs proportion lost to follow-up); otherwise CXR + GX + ECM is more efficient than GX + ECM.

### The effect of population size on efficiency

The effectiveness and efficiency of different strategies in different settings is also influenced by population size. With the mobile unit capable of a fixed number of screening events per year, the larger the population the lower the per-capita rate of screening per year. If the population size and TB burden are low then the absolute number of cases averted is too small for any resource savings to outweigh the intervention resource requirements, making all strategies unfavourable regardless of whether ECM is used (Fig. 5h–k) or not (Fig. 3h–k). Efficiency increases with both increasing population size and TB burden in all the ACF strategies.

In the absence of ECM, simultaneously varying the population size and untreated TB prevalence prior to intervention (all other parameters are fixed to baseline values) shows that the GX option is the most efficient regardless of population size (Fig. 4e). However, in the presence of ECM, if untreated TB prevalence prior to intervention is below 622 per 100,000, then the CXR + GX option replaces the GX option as the most efficient intervention regardless of population size (Fig. 4f). The CXR + GX option is also the most efficient in small population size settings above this TB burden.

### Sensitivity analysis

**Figure 6:**
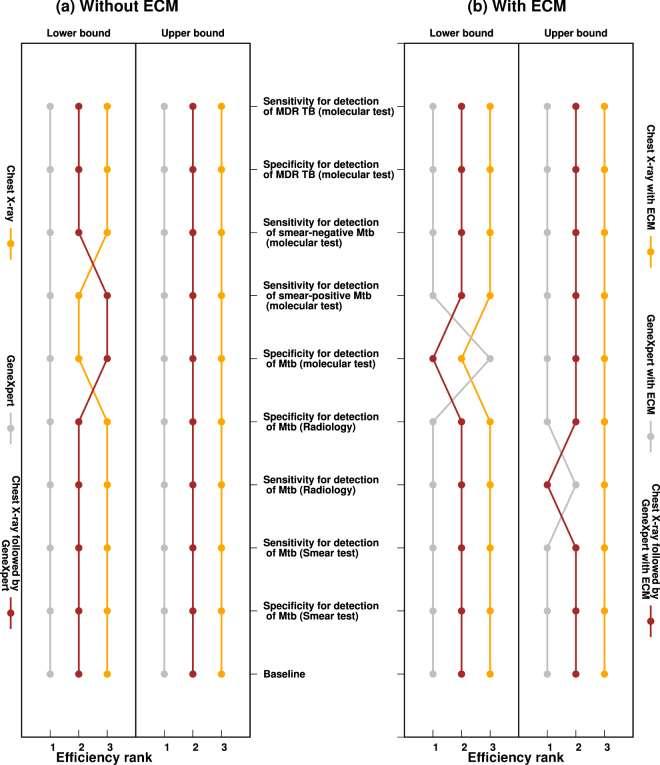
One way sensitivity analysis. Individual parameters were varied by ± 20% from their posterior mean and the rest of the model parameters remained fixed to values in Supplementary Table S1. Model simulations were run with initial untreated active TB prevalence of 1000 per 100,000, of which 7.5% was MDR TB, and each strategy ranked relative to the rest according to efficiency. All individuals screened with GeneXpert wait for their results (no loss to follow-up). (**a**) Shows results from strategies without ECM and (**b**) shows results from strategies with ECM.

We tested the importance of uncertainty in parameter values. The efficiency of the different options is affected by variation in screening and diagnostic accuracy, and this can also affect the efficiency rank order of the options. A decrease in specificity, or sensitivity, lowers the performance and ranking of any ACF strategy using the affected technology due to an increase in false positives, and a reduction true positives, respectively (Fig. 6). An increase in specificity, or sensitivity, has the opposite respective effects. Without ECM, GX is always the top-ranked intervention option regardless of any changes in specificity or sensitivity of the different technologies. In the presence of ECM, the CXR + GX option displaces the GX option as the most efficient intervention in the lower-bound case of GX specificity and in the lower-bound case of sensitivity of CXR. The efficiency rank order of the options remains robust with all other variations in screening and diagnostic accuracy.

## Discussion

We evaluated the effectiveness of different technologies for screening homeless populations for TB, using a mobile unit: chest X-ray (CXR) followed by referral to hospital for diagnosis and treatment if appropriate; GeneXpert (GX) MTB/RIF, which diagnoses TB in those who test positive, in the mobile unit followed by referral to hospital for TB treatment; or CXR followed by GX on the mobile unit followed by referral to hospital for TB treatment for those with abnormal CXR and positive GX results. This study shows that ACF can play an important role in TB control among the homeless, and that it is important to use ECM to ensure high rates of treatment adherence and completion, since inadequate treatment can lead to transmission of TB to others and an increased risk of developing drug resistance in the individual patient.

Whilst other studies have evaluated similar ACF strategies in a general population (e.g. ref.^40^) this is the first study to assess the impact of ACF and ECM strategies targeted at high risk groups with varying TB burden to assess transmission-dynamic effects and efficiency in different settings, which is an important factor when faced with budget constraints. This and other studies in Rotterdam and Paris, showed that ACF in the homeless can reduce TB prevalence over time^5,6^. This was also attributed to a reduction in transmission, as shown by a drop in TB molecular clusters from 75% to 30% in the Paris study^5^, and from 82% to 45% in Rotterdam^6^. Our model accounts for individual-level ACF benefits such as early TB detection and early treatment initiation, as did others^11,40^, but also the model accounts for population-level benefits, in reducing TB transmission.

The most efficient strategy for ACF was generally GX or CXR + GX, depending upon the proportion of patients who are willing to wait the 90 minutes required for GeneXpert testing when it is used as a single-step test. If the proportion lost to follow-up is too high (>27% without ECM or >19% with ECM) then CXR + GX is the most efficient strategy because it greatly reduces the number of patients required to wait for GeneXpert testing, and an abnormal X-ray gives patients an incentive to wait for a TB diagnosis. Empirical research is required to determine the willingness to wait of patients offered single-step GeneXpert screening, and to test our assumption that patients with an abnormal CXR would be willing to wait for their GeneXpert result. Wiliness to wait is likely, however, to be low as demonstrated by a recent UK study which showed that only 8.5% of individuals tested for chlamydia and gonorrhoea were willing to wait over 90 minutes for a point-of-care test result, despite their having sought testing^41^.

The benefit of using GeneXpert in the mobile unit is that it reduces the number of patients who are referred to hospital because only those with TB diagnosed by GeneXpert are referred (for treatment), rather than all patients with abnormal X-ray being referred (for diagnostic testing). It allows for prompt and appropriate treatment initiation, reducing the amount of false positive TB cases. However, screening with GeneXpert excludes a significant number of smear-negative TB cases due to its low sensitivity for such patients, which means that they do not obtain the benefit of treatment, and they remain infectious, although they are less infectious than smear-positive cases^32^.

Sensitivity analysis found that the rank-order of efficiency of different ACF strategies was mainly affected by uncertainty in parameters associated with chest X-ray or GeneXpert diagnostic test sensitivity and/or specificity.

In the ‘lower’ and ‘medium’ TB burden scenarios, efficiency was largely determined by the overall TB burden rather than the ratio of DS TB to MDR TB in the population. High screening and confirmation specificity is particularly important in such settings to reduce false TB positives^40^. These would otherwise incur resource use for screening, hospital referral, and in some cases treatment, for no health benefit. Additional consequences of unnecessary treatment include patient anxiety, morbidity from additional testing possible delays in further diagnostic evaluation^7^. In the ‘higher’ TB burden scenario, high sensitivity for active TB disease is desirable as averted treatment and case management requirements determines efficiency. If the DS TB to MDR TB ratio in a high TB burden population is high then using GeneXpert for TB confirmation can lead to false MDR TB positive cases. Unnecessary treatment with MDR TB drugs is not only resource-intensive, but can also have negative health consequences due to side effects^7^.

In conclusion, active case-finding and enhanced case management of treated patients can reduce TB transmission among the homeless, by reducing the period for which individuals are infectious prior to diagnosis and promoting successful treatment, respectively. Both of these factors reduce the prevalence of active TB cases, which reduces TB transmission, reducing incidence, and thereby reducing future prevalence of disease and need for care. It is important to note that there is a minimum prevalence below which active case-finding is not favourable. Where intervention is favourable, the most efficient approach depends upon the particular setting and upon patient behaviour. A key determinant of whether the most efficient approach is single-step screening with GeneXpert or two-step screening with chest X-ray followed by GeneXpert is the willingness of patients to wait for GeneXpert results, so empirical study is required to assess the feasibility and effectiveness of the two approaches.

## Electronic supplementary material

Supplementary tables and figures
