# Supplementary material for: Comparing different technologies for active TB case-finding among the homeless: a transmission-dynamic modelling study
Source: Sci Rep. 2018 Jan 23;8:1433. doi: 10.1038/s41598-018-19757-5 (PMC5780390; doi:10.1038/s41598-018-19757-5)
Supplement: Supplementary file 1 — Supplementary tables and figures [file 41598_2018_19757_MOESM1_ESM.pdf]

Comparing different technologies for active TB case-finding among the homeless: a transmission-dynamic modelling study.

Tendai Mugwagwa[1,2,*], Helen R Stagg[3], Ibrahim Abubakar[3,4], Peter J White[1,2]

[1]Modelling and Economics Unit, National Infection Service, Public Health England, London, UK

[2]MRC Centre for Outbreak Analysis and Modelling, and NIHR Health Protection Research Unit in Modelling Methodology, Department of Infectious Disease Epidemiology, Imperial College London, UK

[3]Institute for Global Health, Faculty of Population Health Sciences, University College London, UK

[4]Medical Directorate, Public Health England, London, UK

CORRESPONDING AUTHOR: Dr Tendai Mugwagwa, Modelling and Economics Unit, National Infection Service, Public Health England, 61 Colindale Avenue, London, NW9 5EQ, UK. E-mail: tendai.mugwagwa@phe.gov.uk.

SUPPLEMENTARY INFORMATION

Supplementary Table S1: Default transmission model parameter values and data sources.

**Parameter description**	**Default value / range**	**Source / Reference**
Population characteristics		
Size of population	20,000<br>Range: 5,000-25,000	[12]
Rate of exit from the population	0.144 p.a.<br>Range: 0.1-0.25	[18]
Untreated active TB prevalence per 100,000	100-2,000	Range examined
Percentage of TB that is MDR at baseline	2%-15%	Range examined
TB natural history		
Percentage of incident infections that are slow-progressing	86%	[16]
Per-capita rate of slow progression to active TB disease	1.13×10^{-4} p.a.	[16]
Per-capita rate of fast progression to active TB disease	0.88 p.a.	[16]
Percentage of new disease that is smear positive	45%	[16]
Per-capita mortality rate of untreated active disease	0.23 p.a.	[19]
Per-capita rate of conversion from smear-negative to positive	0.015 p.a.	[16]
Per-capita rate of self-cure: natural reversion from active disease to latent infection	0.21 p.a.	[19]
Relative transmissibility of MDR TB compare to DS TB	Fitted	Fitted for each scenario
Probability of acquiring MDR TB after treatment failure	3.5%	[20]
Screening & treatment		
Per-capita rate of passive case-finding	3.02 p.a.	[11]
Per-capita rate of active case-finding by Mobile Unit	0.78 p.a.	[11]
Percentage of general population DS TB cases treated successfully without ECM	85%	[1]
Percentage of general population MDR TB cases treated successfully without ECM	75%	[1]
Percentage of homeless DS TB cases treated successfully without ECM	46%	[11]
Percentage of homeless MDR TB cases treated successfully without ECM	33%	Based on national average,[1]
Mean duration of successful DS treatment	6 months	[13]
Mean duration of unsuccessful DS and RIF-R/MDR treatment	2 months	[21]
Mean duration of successful MDR treatment	20 months	[13]
Per-capita mortality rate of unsuccessfully treated disease	0.077 p.a.	[16]
Time to culture and DST result	28 days	[7]

Transmission		
Transmission parameter for smear-positives	Fitted	Fitted
Relative infectivity of smear-negatives (vs. smear-positives)	0.22	[17]
Relative susceptibility of Latent (slow) and Recovered patients (vs. susceptibles)	0.35	[16]
Relative infectivity of unsuccessfully treated with appropriate regimen (vs. untreated)	0.25	[16]
Relative infectivity of treated with inappropriate regimen (vs. untreated)	1	Assumed
Test performance		
GeneXpert TB sensitivity if patient is smear negative	73%	[22]
GeneXpert TB sensitivity if patient is smear positive	98%	[22]
GeneXpert TB specificity if patient is smear negative or positive	99%	[22]
Specificity of GeneXpert in identifying MDR TB	98%	[22]
Sensitivity of GeneXpert in identifying MDR TB	98%	[22]
Chest X-ray TB specificity	63%	[15]
Chest X-ray TB sensitivity	73%	[15]
Sputum smear microscopy TB specificity	77%	[23]
Sputum smear microscopy TB sensitivity	53%	[23]

Supplementary Table S2: Economic parameter values and data sources.

Parameter	Value	Reference
Discount rate	3.5% p.a.	[14]
Treatment cost: DS TB	£4,940	[11,24]
Treatment cost: MDR TB	£15,683	[24]
Clinical examination costs	£278	[24]
Assisted referral to clinic cost	£55.48	[25]
Diagnostic culture test cost	£22.29	[13]
Diagnostic GeneXpert test cost	£38.83	[27]
Smear microscopy cost	£7.64	[11]
Annual Mobile Radiology Unit intervention cost	£568,909	[11]
Mobile Radiology Unit capital cost	£644,019	[11]
Digital radiography machine cost	£125,275	[26]
GeneXpert purchase cost (16-channel)*	£145,570	[27]
Annual GeneXpert maintenance cost (16-channel)*	£8,528	[27]
GeneXpert purchase cost (4-channel: 1/4 cost of 16-channel)*	£36,392	[27], [28].
Annual GeneXpert maintenance cost (4-channel: 3/8 cost of 16-channel)*	£3,198	[27],[28].

*Including (20%) Valued Added Tax

Supplementary Table S3: QALY parameter values and data sources.

Health state	QoL weight	Reference
Uninfected population	0.87	[29]
Any untreated active TB	0.68	[30]
Active TB on DS TB treatment	0.81	[30]
Active TB on MDR TB treatment	0.68	[31]

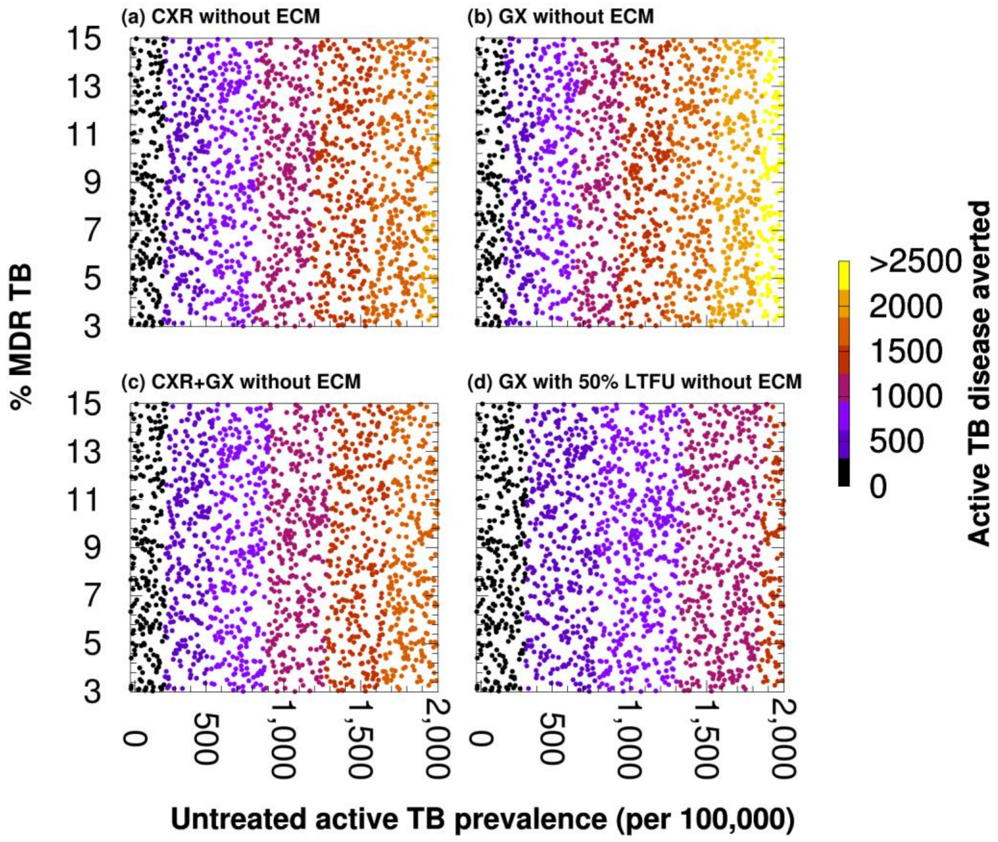

Supplementary Figure S1: Active TB disease averted by different active case finding (ACF) strategies without enhanced case management in settings with varying TB burden. The impact of different ACF strategies on transmission dynamics were compared with current practice in settings with different TB prevalence and proportion that is multi-drug/rifampicin resistant (MDR). (a-d) The incremental number of averted active TB disease cases was determined over the lifetime of the population cohort. (b,c) All individuals screened with GeneXpert wait for their results (no loss to follow-up) except for (d) which assumes the GeneXpert only option with a 50% loss to follow-up.

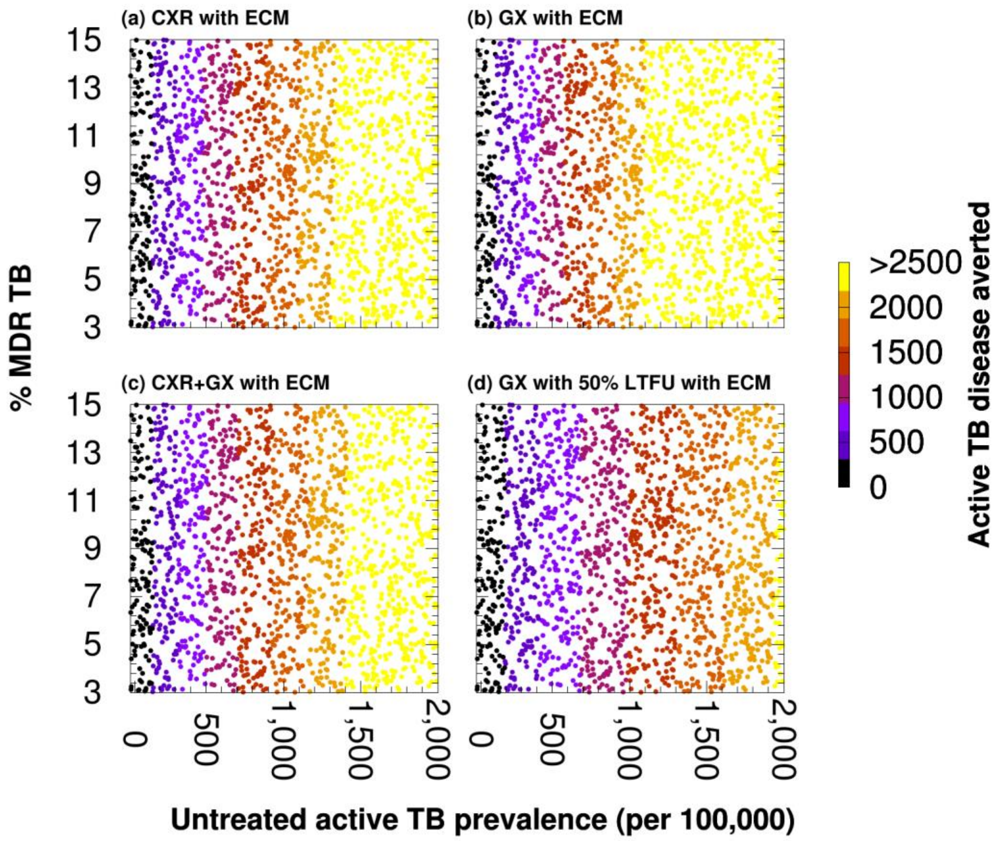

Supplementary Figure S2: Active TB disease averted by different active case finding (ACF) strategies with enhanced case management in settings with varying TB burden. The impact of different ACF strategies on transmission dynamics was compared with current practice in settings with different TB prevalence and proportion that is multi-drug/rifampicin resistant (MDR). (a-d) The predicted incremental number of averted active TB disease cases was determined over the lifetime of the population cohort. (b,c) All individuals screened with GeneXpert wait for their results (no loss to follow-up) except for (d) which assumes the GeneXpert only option with a 50% loss to follow-up.
